# Clinical Analysis of 137 Cases of Ovarian Tumors in Pregnancy

**DOI:** 10.1155/2022/1907322

**Published:** 2022-05-25

**Authors:** Qi Yin, Min Zhong, Zhihui Wang, XiuJie Sheng

**Affiliations:** Department of Obstetrics and Gynecology, Center for Reproductive Medicine/Department of Fetal Medicine and Prenatal Diagnosis, Key Laboratory for Major Obstetric Diseases of Guangdong Province, The Third Affiliated Hospital of Guangzhou Medical University, Guangzhou, 510150 Guangdong, China

## Abstract

Ovarian tumors do not really typically occur in association with pregnant; however, once they do, the treatment is critical. It is important to note that around 6% of ovarian tumors in pregnancies are cancerous. The problems induced by ovarian tumors in pregnancy particularly necessitate rapid medical intervention and are much more frequent than cancer. Medication choices and survival of ovary tumor patients could be influenced by varied diagnoses of ovarian masses. So, we present an upgraded logistic regression (ULR) approach in this paper. Initially, the collection of 137 patient datasets was employed in screening test to identify the ovarian tumor as benign-tumor and malignant-tumor by using contrast-enhanced ultrasonography (CEU) method. Then, the screening test images are preprocessed using wavelet transform (WT) approach. The preprocessed data are extracted by using local binary pattern (LBP) and laws' texture energy (LTE) techniques. Finally, the clinical analysis of the ovarian tumor can be obtained by the proposed ULR approach. The performances were examined and compared with existing approaches to achieve the proposed approach with greatest correctness. The findings are depicted by utilizing the MATLAB tool.

## 1. Introduction

Ovarian tumor is among the most prevalent cancers in women's healthcare systems worldwide. Epithelial ovarian tumor is the most common pathologic kind, and it was identified in approximately 10 percent of people under the age of 40. Among teenage people with acute ovarian tumor, pregnancy protection is critical [1]. Every cancerous formation in the ovary is referred to as an ovarian tumor. Ovarian malignancies are most commonly caused by the outside layer of the ovary. It causes abnormal cells to form, which could also damage or spread throughout the body. There seem to be no or very minor negative effects whenever this begins. As the development process advances, negative effects become increasingly noticeable. Bloating, pelvic pain, stomach swelling, and a lack of appetite may occur as a result of these issues [2].

Ovary illness, the seventh most common malignant in women, is indeed the 5th leading cause of death in women and the deadly of gynecologic cancers. Each female's likelihood of developing invasive ovarian cancer was 1 per 75, while her future chance of death from malignant ovarian cancer becomes 1 per 100. Moreover, females between the ages of 54 and 65 are more likely to get ovarian cancer. Usually, a 5-year survival time has been used to evaluate kinds of cancer, and health outcomes vary depending on the course of the illness [3]. Female who are diagnosed early, before the tumor has progressed, have a significantly greater 5-year survival rate than those who are diagnosed later. The portion of people suffering from cancer is increasing as a result of the delay caused by arranging pregnancy at a late productive time and the fact that the incidence of the development of multiple cancers rises throughout the fifth decade of life. This difficult disease affects one in every thousand pregnancies and has become increasingly widespread in recent years. Adnexal tumors are known to occur in 0.15–5.7 percent of pregnant women, with the majority of them would be healthy.

In the healthcare profession [4], there has been a significant advancement in ultrasound scans, especially performs a critical function. Image analysis is a method for separating important areas in an optical image. Splitting has dealt with a lot of diagnostic concerns, as well as the repercussions of many of these problems, and this is an important investigation area that really has drawn basic scientists' attention in the past, prompting many techniques in diverse directions of division. Focused mainly on, a whole image of an ovary malignancy is the object of separation into cancerous borders and placement, as well as little important segments to more easily investigate the disease.

In ovarian cancer patients, CEU [5] is largely considered as a valid and efficient diagnostic testing tool. CEU uses micro bubble contrast agents can disclose critical and sensitive data regarding tissue perfusion including blood supply. It is also very useful for viewing tiny arterial arteries with diameters less than 100 m. CEU is indeed a generally secure procedure that does not use radiation exposure or pose a danger of nephrotoxicity. Despite color Doppler ultrasonography, which would be capable of assessing vast vascular systems in tumors, CEU performs excellent at investigating tumor microvascularity that is related with modest blood movements having similar acoustic qualities making CEU a significant benefit in ovarian cancer cases. CEU significantly increases the identification of microcirculation perfusion, and earlier researches have shown that the MVD variation is useful in determining whether an ovarian cancer appears normal or abnormal.

The ultrasonographic ovarian image is preprocessed to reduce noise present and enhance the maximum signal to noise ratio enabling ovarian tumor perfection and correct identification. The preprocessed ovarian ultrasonography picture is then delivered to the tumor image retrieval module, where it is used to discriminate properties such as area, shape, and shading. Some females are indeed recommended treatment since they are concerned about likelihood of cancer that is both pricey and risky. When a mathematical analysis could effectively determine the likelihood of malignant in women having ovarian tumors based on basic diagnostic and ultrasonographic characteristics, it would have been useful for counseling, as well as identifying the best surgical strategy, incision form, and treatment.

This research presents an upgraded logistic regression (ULR) approach for clinical assessment. Here, 137 patient's datasets are utilized and are performed in scanning test for identification of the ovarian tumor like benign and malignant. Then, the identified images are collected and are preprocessed using WT approach. Next, two approaches (LBP and LTE) are utilized for extracting the characteristics of the preprocessed data. Then, the clinical examination was carried out by the proposed approach. Finally, the performances of the proposed approach are examined to obtain this work with greater effectiveness. The outcomes are depicted by utilizing the MATLAB environment. The additional part of this work can be divided as follows: Section 2 provides the related works and problem statement; Section 3 demonstrates the proposed work; Section 4 depicts the results and discussion; Section 5 summarizes the full research.

## 2. Related Works

Preoperative examination of adnexal masses can be done using a variety of therapeutic tests [6]. Modern IOTA methodologies including the Simple Rules and also the ADNEX framework, based on predetermined concepts and evaluation methods, are indeed the greatest of existing options, as evidenced by direct comparisons using IOTA phase iii information and comprehensive studies including meta-analysis. ADNEX has been the first multiclass forecasting model, providing hazard maps for several kinds of cancers. It does have the greatest result in distinguishing from malignant and benign. Even as incident studies demonstrate, this is extremely important in guiding treatment outcome [7]. They established beneficial results for child and the parents despite extensive chemotherapy and radiotherapy while pregnant following multidisciplinary physicians' instructions. It was the first example of docetaxel as well as carboplatin combined treating tumors in pregnant. Aside from the difficulty of saving the fetus while curing the mom, the therapy principles of maximal debulking surgery and chemotherapy in susceptible individuals are nearly identical. Surgical preservation of the uterus, teratogenicity of chemotherapy medicines, but also long-term adoption all seem to be specific to ovarian cancer throughout pregnant. When females delaying children, the incidence of cancer during pregnant rises, posing a huge medical and social dilemma affecting culture and way of life. Additional instances and follow-ups are required for conventional therapy techniques [8]. The “microcystic structure” comprising BOT papillary extensions, solid elements, and/or septa is described. All serous and mucinous BOT patients have these. The microcystic structure seems to be specific to BOTs, based on the similarity of sonographic pictures and histology of malignant and benign tumors. No prior review article reported, described, and illustrated the established sonographic features of BOTs. With this novel marker, BOTs could be differentiated from ovarian malignancies and benign ovarian diseases, allowing for proper specialized medical therapy [9]. Resulting in increasing chance of tumor recurrence, the birthrate during BOT fertility-sparing treatment was acceptable, with really no influence on patient survival. Recurrence as well as pregnant has also been duration independent, with such a proportional probability throughout adoption [10]. This method is useful for identifying remote as well as nodal metastases when conjunction with standard imaging technologies. WB-DWI/MRI might eliminate the requirement for irradiation, color insertion, or several screening procedures while pregnant. This reduces oncological stage, timekeeping, financial implications, and fetus exposure to radiation, as well as patient mental anguish [11]. The findings show the laying hens naturally form polycystic ovary disease that is linked to chronic inflammatory diseases. Inflammatory and chronic stress were characteristics of malignant growth; hence, PCO is also an ovarian cancer significant predictor. This strategy focuses on the discovery of PCO hens that subsequently evolved OVCA. Therefore, the laying hen could be used to study the origin of random polycystic ovary disease as well as its lengthy threat of ovary tumor progression. The concept could also be used to identify techniques to avoid polycystic ovary disease and OVCA growth [12]. Based on the initial report, semiquantitative DCE-MRI can detect cancerous from normal as well as borderline of benign lesions, however not between the two, despite the distinct TIC kind. Additional research integrating statistical DCE-MRI, magnetic resonance spectroscopy, with diffusion-weighted scanning, is recommended [13]. Adding CESM to the process requires just modest changes to existing apparatus and personnel training. They also discovered that CESM improves ultrasonography yield as well as detects mammographically concealed breast tumors. Only significant contrast allergies, renal disease, and restricted intravenous accessibility were contraindications. Nevertheless, comparing aberrant augmentation to focused scanning enables for a reliable identification, particularly when the tumor upon this ultrasound was harmless [14]. A higher recurrence probability and shorter DFS were related with fertility sparing treatment than surgical intervention in this research. So, fertility sparing therapy must be advised with care. Stage II, serous kind, bilateral, and micropapillary tumors are related with a greater recurrence with shorter recurrence interval following conservative treatment, according to this study's retrospective study. Individuals having borderline ovarian tumors must avoid fertility-saving treatment and try to conceive as quick as practicable. Individuals who are not prepared to conceive could be directed to regenerative endocrinology and infertility hospitals for further fertility alternatives such as storing embryos, oocytes, and other reproduction components [15]. There have been no randomized control trials specifically compared the impact of laparoscopy for harmless ovarian tumors in pregnant upon mother as well as fetal health or treatment adherence [16]. They used ROC curves as well as regression analysis to identify the optimum mixtures of 5 serological tests to cancer screening in diverse communities. These findings imply the integrating ROC contour as well as logistic statistical modeling to detect tumor markers was viable [17]. While certain morphological as well as quantitative distinctions existed between the BOT and MEOT categories, such differences were not adequate to differentiate between the two gatherings. Therefore, they created diagnostic algorithms using variable combinations that distinguish BOTs against MEOTs. For my understanding, nobody has considered separating BOTs and MEOTs utilizing multivariate logistic test to predict the most important MR data. The multivariate logistic analysis demonstrated that ADCmin and maximum solid element diameter have been distinct MEOT markers. Unlike ADC assessment, CA-125 would not be a good determinant for distinguishing BOTs and MEOTs [18, 19].

### 2.1. Problem Statement

Ovarian tumors typically occur in pregnancies; however, if they do, treatment is critical. Furthermore, around 6% of ovarian tumors in pregnancies are cancerous. Pregnancy complications induced by ovarian tumors require immediate medical attention and are much more frequent than cancer. Several ovarian mass diagnoses could influence ovary tumor patients' medication choices and survival. So, in this paper, we propose upgraded logistic regression (ULR). Initially, 137 patient datasets are being used to screen for benign and malignant ovarian tumors using contrast-enhanced ultrasonography (CEU). Then, the screening test images are wavelet transform (WT). The preprocessed data are extracted using LBP and LTE techniques.

## 3. Proposed Work

**Figure 1:**
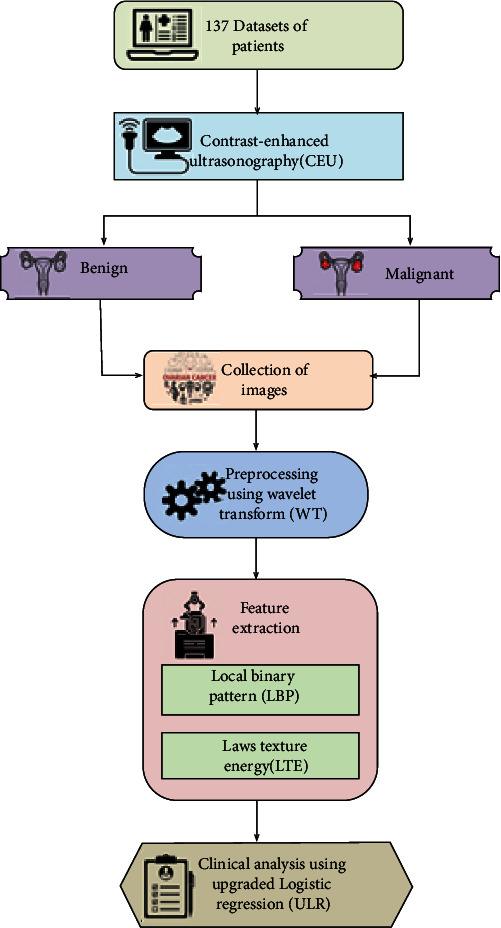
Structure of the proposed work.

In this phase, we demonstrate the proposed ULR approach for clinical assessment of the ovarian tumor while pregnancy. Here, 137 patient's datasets are utilized and are performed in scanning test for identification of the ovarian tumor like benign and malignant. Then, the identified images are collected and are preprocessed using WT approach. Next, two approaches (LBP and LTE) are utilized for extracting the characteristics of the preprocessed data. Then, the clinical examination was carried out by the proposed approach.  Figure 1 outlines the structure of the proposed research.

### 3.1. Patient's Dataset

The clinical data of 137 patients with pregnancy complicated with ovarian tumor who were hospitalized in the Third Affiliated Hospital of Guangzhou Medical University from 2008 to 2019 were collected. The following have been the article's requirements: (1) woman with a definitive histological diagnosis for MOGCT/OSCST, (2) woman who seemed to have FSS at such two major hospitals and also had a full clinical history, and (3) woman underneath the age 40 who gave permission and then were available for join. Patient documents, surgical and pathological files, and chemotherapy files were used to collect demographic and clinical facts from sufferers' health files. The age at the time of testing, the phase of the illness, the day and kind of treatment, the chemotherapy protocol, and the cycle counts were all collected.  Table 1 depicts the clinical characteristics of 137 cases.

### 3.2. Contrast-Enhancement Ultrasonography (CEU)

According to the CEU procedure, the sonography has been conducted transabdominally (TA) among 137 patients. The complete 180-second evaluation is documented and afterwards assessed by a physician and a gynecologist. The variables that had been investigated were as follows:

Examination of morphology with/without contrast enhancement is as follows: (1) no supplemental data beyond what normal sonography provides, (2) data that does not even change the way the tumor is managed, and (3) data that influences tumor care (wait-and-see vs. treatment, operation schedule, and operative approach being used and aim vs. treatment).

Graph of time/intensity is as follows: (1) highest augmentation time and (2) highest augmentation intensity represented as 3 distinct measures assessed at 3 distinct locations in the ovarian tumor.

Beginning with the initial presentation of contrast balloons inside the visual field, the time/intensity graphs have been assessed. Finally, we obtain the dataset in forms of pictures as benign tumor and malignant tumor for image preprocessing phase.

### 3.3. Preprocessing Using Wavelet Transform (WT)

Regarding pictures with the least abstract level, preprocessing has been used. Preprocessing step can be used to enhance a picture via eliminating mask or boosting certain picture qualities that are important for later handling. The goal of a picture noise removal technique is always to remove noise while preserving features. Throughout picture capture and processing, there is indeed a risk of picture degradation due to noise. Denoising a picture eliminates additive noise such as pepper and salt, dots, and Gaussian noise while preserving essential image properties.

Denoising a picture has been done with the wavelet transform. Wavelet transform shows the typical information of a picture including its geographical location. Inside the wavelet domain, distortion was evenly distributed among coefficients, while the majority of picture information usually focused throughout a few big ones.

**Figure 2:**
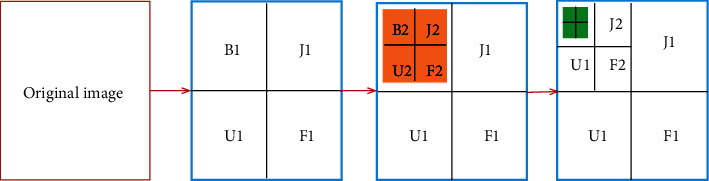
Structure of 3-stage WT decomposition.

For B1, J1, U1, and F1, the picture would be split into the first stage via wavelet decomposition. Within next stage, it would be separated into 2 stages of wavelet decomposition, with section B1 being further separated into B2, J2, U2, and F2. Lastly, the three-level decomposition section B2 would be divided as a WT3-stage decomposition. Following that, the picture would be split into 3 stages of WT decomposition for extraction phase.  Figure 2 depicts the structure of 3-stage WT decomposition.

### 3.4. Feature Extraction Using Local Binary Pattern (LBP) and Laws' Texture Energy (LTE)

A geographic type of dimension mitigation seems to be extraction of features. LBP and LTE approaches were employed in this phase.

#### 3.4.1. Local Binary Pattern (LBP)

**Figure 3:**
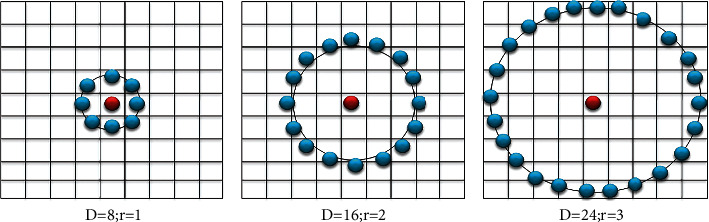
Circularly symmetric neighbor arrangements for various *D* and *r* amounts.

LBP has been presented as a texture processor that handles picture pixels via thresholding its (3 × 3) neighborhood as well as converting the obtained tag to a binary form. As a textural attribute, the histogram among these tags has been employed. The LBP operator then was expanded to include neighborhoods of varied sizes. For texture separation to facial identification, LBP has really been effectively widely used in a number of scenarios. The various stages are used to produce the LBP feature representation:

*Stage 1*. Take a pixel's circular neighborhood. *D* dots on the perimeter of a circle having radius *r* that are mostly equal from central pixel are picked. Let *g*_*c*_ become the centre pixel's grey-level intensity value. The grey-level intensity amounts of the *D* locations are *g*_*D*_, *D* = 0, ⋯, *D*–1.  Figure 3 shows circularly symmetric neighbor arrangements for various *D* and *r* amounts.

*Stage 2*. Such *D* points are transformed into a circle bit-stream comprising zeroes and ones based upon whether pixel's intensity will be smaller than or higher than the centre pixel's intensity. The below formula is used to determine the amount of the LBP pattern of the central pixel (*x*_*c*_ and *y*_*c*_) having intensity *g*_*c*_.

(1) $$\begin{array}{c} {\text{LBP}}_{D,r}^{\text{orig}}=\overset{D-1}{\underset{D=0}{\sum }}T\left(g_{D}-g_{C}\right). \end{array}$$

Here,

(2) $$\begin{array}{c} T\left(x\right)=\left\{\begin{array}{cc} 1, & x\geq 0,\\ 0, & x<0. \end{array}\right. \end{array}$$

The term of uniformity was premised on the reality that certain binary sequences appear more frequently than others. The texture descriptor was subsequently computed using regular pixels, which are classed as uniform or nonuniform. The geographical transformations *U* (quantity of geographical bit-wise 0/1 transformations) in such uniform basic designs are quite minimal. Whenever a binary sequence is traversed circularly, the LBP was said to have been uniform whether there are no more than two bitwise transformations from 0 to 1 or conversely.

A rotation invariant using uniformity amount can be determined depending upon the amount of transformations in the neighbor design.

(3) $$\begin{array}{c} {\text{LBP}}_{D,r}\left(x\right)=\left\{\begin{array}{cc} \overset{D-1}{\underset{D=0}{\sum }}T\left(g_{D}-g_{C}\right) & \text{if }U\left(x\right)\leq 2,\\ D+1 & \text{otherwise}. \end{array}\right. \end{array}$$

Here,

(4) $$\begin{array}{c} T\left(x\right)=\left\{\begin{array}{cc} 1, & x\geq 0,\\ 0, & x<0. \end{array}\right. \end{array}$$

The picture is multiscaled employing LBP via selecting circles having different radii around the central pixels and afterwards creating an individual LBP image to every dimension. The energy as well as entropy of the LBP picture, generated at various sizes, also used as characteristic descriptors.

#### 3.4.2. Laws' Texture Energy (LTE)

Various masks of relevant dimensions are considered useful in differentiating among various varieties of texture, according to laws. This technique works by assigning such masks to a picture and afterwards calculating the energy inside the filter pass domain. The texture energy measurements are calculated employing three 1-dimensional vectors: L3, B3, and D3, which represent the level, border, and dot characteristics, correspondingly.

When every vertical 1-dimensional vector is integrated with such a perpendicular one, 9 2-dimensional masks with dimension 3 × 3 have been created, specifically L3L3, L3B3, L3D3, B3B3, B3L3, B3D3, D3D3, D3L3, and D3B3.

(5) $$\begin{array}{c} \begin{array}{c} \left(\begin{array}{c} 1\\ 2\\ 3 \end{array}\right)*\begin{array}{c} \left(-1\;0\;1\right)\\ B3 \end{array}=\begin{array}{c} \left(\begin{array}{ccc} -1 & 0 & 1\\ -2 & 0 & 2\\ -1 & 0 & 1 \end{array}\right)\\ L3B3 \end{array}.\\ L3 \end{array} \end{array}$$

Exceptions of L3L3, each of these masks gets a zero mean. Those eight zero-sum masks, ranged 1 to 8, were employed. The picture *P*(*i*, *j*) has been initially combined with every two-dimensional mask for retrieve texture information. By instance, when we are using B3B3 to filter the picture *P*(*i*, *j*), the output becomes as

(6) $$\begin{array}{c} {TP}_{\text{B}3\text{B}3}=P\left(i,j\right)*\text{B}3\text{B}3. \end{array}$$

The texture picture *TP*_L3L3_ had been used to balance the intensity of the other texture pictures, as given in below equation, and produce the output images contrast-free.

(7) $$\begin{array}{c} \text{Balance}\left({TP}_{\text{mask}}\right)=\frac{{TP}_{{\left(i,j\right)}^{\text{mask}}}}{{TP}_{{\left(i,j\right)}^{L3L3}}}. \end{array}$$

Texture Energy Metric (TEM) filters have been applied to the balanced pictures that comprised of a moving nonlinear frame mean of real numbers.

(8) $$\begin{array}{c} {\text{TEM}}_{\left(i,j\right)}=\overset{3}{\underset{u=-3}{\sum }}\overset{3}{\underset{v=-3}{\sum }}\left|{TP}_{\left(i+u,j+v\right)}\right|. \end{array}$$

As a result, the picture during examination was filtered employing all these 8 masks, while respective energies were calculated and employed as descriptors throughout the extraction phase.

### 3.5. Upgraded Logistic Regression (ULR) Analysis

LR analysis expands multiple regression techniques for investigation circumstances when the result variable is categorical. Classified results are extremely usual. Assessments of gain/loss or enhanced/not-enhanced outcomes can be formed while assessing training materials. In a hospital field, a result could be illness existence or disappearance.

By concept, a continuous result parameter is indeed a linear combination of a collection of predictor variables as well as error, according to the core idea underpinning multiple regression test (MRT). The MRT approach takes the following form for a resultant parameter, *R*, as well as a collection of “*k*” predictors, *M*_1_, ⋯, *M*_*k*_:

(9) $$\begin{array}{c} R=\alpha +\beta_{1}M_{1}+\beta_{2}M_{2}+\cdots +\beta_{k}M_{k}+\varepsilon =\alpha +\overset{k}{\underset{j=1}{\sum }}\beta_{j}M_{j}+\varepsilon . \end{array}$$

Here, *β*_*j*_ is the multiple regression factor and *ε* is the prediction error.

If error is not included, the modeling that results indicates the predicted result.

(10) $$\begin{array}{c} E\left(R\left|M_{1,\cdots .,}M_{k}\right.\right)=\overset{´}{R}=\alpha +\overset{k}{\underset{j=1}{\sum }}\beta_{j}M_{j}. \end{array}$$

Keep in mind that *R* = *R*′ + *ε*.

As a result, the MRT concept could be interpreted as follows: Every measured value, *R*, is determined by two factors: a predicted element, *R*′, which would be a consequence of the predictive variables *M*_1_, ⋯, *M*_*k*_, and then an error (unpredictable) element, which indicates testing error (i.e., inaccuracy).

The MRT instructed above can be used when the outcome variable, *R*, was continual, but it could be used whenever *R* was categorical.

When *R* is set to 1 for “gain” and 0 for “loss,” the multiple regression formula will not produce predicted scores that are either precisely 1 or 0.

Whenever a result variable is categorical, the approach for ULR provided following would be a more realistic depiction.

The result parameter, *R*, is assumed to be categorical in the ULR; however, ULR does not properly represent that result parameter. ULR is instead predicated on the probabilities related to *R* values. ULR has been focused on a linear model for the natural log of such probabilities with favor of *R* = 1 on conceptual and quantitative purposes.

Throughout the domain of regression, this is considered that a collection of predictor parameters were associated to *R* and then bring extra data to predicting *R*.

(11) $$\begin{array}{c} \log_{e}\left[\frac{\left.P\left(R=1\right)\left|M_{1},\cdots ,M_{k}\right.\right)}{1-P\left(R=1\left|M_{1},\cdots ,M_{k}\right.\right)}\right]=\log_{e}\left[\frac{\pi }{1-\pi }\right]=\alpha +\beta_{1}M_{1}+\cdots +\beta_{k}M_{k}=\alpha +\overset{k}{\underset{j=1}{\sum }}\beta_{j}M_{j}. \end{array}$$

Here, *π* is the conditional probability of {*P*(*R* = 1|*M*_1_, ⋯, *M*_*k*_)}.

Inverse transformation is as follows:

(12) $$\begin{array}{c} P\left(R=1\left|M_{1},\cdots ,M_{k}\right.\right)=\frac{e^{\alpha +\sum_{j=1}^{k}\beta_{j}M_{j}}}{1+e^{\alpha +\sum_{j=1}^{k}\beta_{j}M_{j}}}. \end{array}$$

Because of the quantitative relation (13), the logistic expression for ULR will be the following:

(13) $$\begin{array}{c} \frac{e^{a}}{\left(1+e^{a}\right)}=\frac{1}{\left(1+e^{-a}\right)}, \end{array}$$

(14) $$\begin{array}{c} P\left(R=1\left|M_{1},\cdots ,M_{k}\right.\right)=\frac{e^{\alpha +\sum_{j=1}^{k}\beta_{j}M_{j}}}{1+e^{-\alpha -\sum_{j=1}^{k}\beta_{j}M_{j}}}. \end{array}$$

Because of the quantitative formula (15), the probability of a “zero” response will be the following:

(15) $$\begin{array}{c} 1-\frac{e^{a}}{\left(1+e^{a}\right)}=\frac{1}{\left(1+e^{a}\right)} \end{array}$$

(16) $$\begin{array}{c} P\left(R=0\left|M_{1},\cdots ,M_{k}\right.\right)=1-P\left(R=1\left|M_{1},\cdots ,M_{k}\right.\right)=\frac{1}{1+e^{\alpha +\sum_{j=1}^{k}\beta_{j}M_{j}}}. \end{array}$$

## 4. Results and Discussion

In this phase, we are going to explain the clinical analysis of the ovarian tumor in pregnant time employing the proposed ULR approach. The findings of the proposed approach are depicted by utilizing the MATLAB tool. Here, totally 137 cases/patients recorded. Analytical importance has been defined as a *P* value of less than 0.05.  Table 2 outlines the ULR analysis of 137 ovarian cases. Totally, there 64 cases of border-line ovarian tumor (BOT), and that includes 38 serous, 22 mucinous, 2 endometrioid, 1 seromucinous, and 1 other type of the cancer. Similarly, there are 28 cases of epithelial ovarian cancer (EOC), and that includes 12 serous, 11 mucinous, 2 primary peritoneal, 2 clear cell types, and 1 Brenner type. Totally, there are 32 cases of malignant ovarian germ cell tumors (MOGCTs), and that includes 14 immature teratoma, 16 dysgerminoma, and 2 strumal carcinoid types. Totally, there are 4 cases of malignant sex cord-stromal tumors (MSCTs), and that includes 2 Sertoli-Leydig tumors and 2 granulosa cell tumors. Then, there are 2 small cell carcinoma (SCC) cases. Then, overall, there are 7 cases of metastatic carcinoma, and that includes 4 Krukenberg tumors, 2 cervix mixed adenocarcinoma, and one B-cell lymphoma type.

As per pregnant results, the sufferers are split into 2 groups: abortion (32 cases) and live birth (105 cases).  Table 3 compares the medical characteristics of the two categories. Fifty-two cases were identified with malignant tumors at the 3rd trimester (196 days) of pregnancy gave birth to healthy babies. The quantity of cases with ovarian tumors found in prepregnancy, 1st trimester, and 2nd trimester throughout the abortion category were 22 and 10 correspondingly. The quantity of cases with ovarian tumors found in prepregnancy, 1st trimester, and 2nd trimester throughout the live-birth category were 22 and 10 correspondingly. The numbers of malignant tumors detected during distinct gestational-age (GA) levels throughout the live-birth category comprised 8, 42, and 55 cases with a statistical significance variation (*P* < 0.05). Throughout the live-birth category, 101 cases had treatment throughout the 2nd and 3rd trimesters of pregnancy: 19 patients had treatment during 2nd trimester, and 82 patients had treatment during 3rd trimester. Over 50% of the abortion cases (21 patients) had treatment in the 1st trimester.

Due to the trend of waiting birth to next reproductive ages including the use of approved reproductive methods, the identification of cancerous ovarian tumors throughout pregnant seems to be on the upswing. Throughout the statistics provided above, BOT has been the most common ovary malignant among pregnant ladies (64 cases), next by EOC (28 cases), and germ cell tumors (32 cases). This pattern is similar to that shown in earlier studies.

The treatment for ovarian masses while pregnant is identical to that of nonpregnancy, including maternal-fetal variables taken into account. Therapy must be tailored to the disease kind, phase, gestational age, and preferences of the mother. Within second trimester of pregnancy, treatment, a core of intervention for cancerous ovarian tumors, was advised to minimize the incidence of abortion, twisting, displacement, and late identification of malignant. Complete surgical phasing is one of the quality management. Restaging operation at caesarean section or postdelivery could be necessary to decide the care plan whereas if pelvic peritoneum as well as the pouch of Douglas cannot be properly evaluated at operation.

BOTs get a high success rate, and the majority of cases have been managed surgically rather than with chemo-treatment. Regarding women of reproductive age having stage I malignancies, fertility-sparing treatment is favored. Restaging treatment does not really reduce reappearance in cases with BOTs, according to a meta-analysis, and it introduces cases in additional anesthetic and operational risks. Our data suggest that pregnancies having ovarian tumor problems have generally favorable results.

### 4.1. Accuracy (acc)

It is specified by the proportion of successfully identified cases to the total cases. In this phase, performances like accuracy, precision, recall, and f1-score are examined on the support of (tp: true positive, tn: true negative, fp: false positive, and fn: false negative) and that findings are depicted by employing the MATLAB environment.

(17) $$\begin{array}{c} \text{acc}=\frac{\left(tp+tn\right)}{\left(tp+tn+fp+fn\right)} \end{array}$$

Here, tp (true positive) indicated the patients who have been appropriately identified as having malignant nodes, indicating that they have the illness; tn (true negative) is the amount of accurately identified healthy persons; fp (false positive) counts the set of patients who have been incorrectly classified as having the disease while they are completely healthy; fn (false negative) specifies the number of patients who are mistakenly labeled as healthy when they are truly sick.

**Figure 4:**
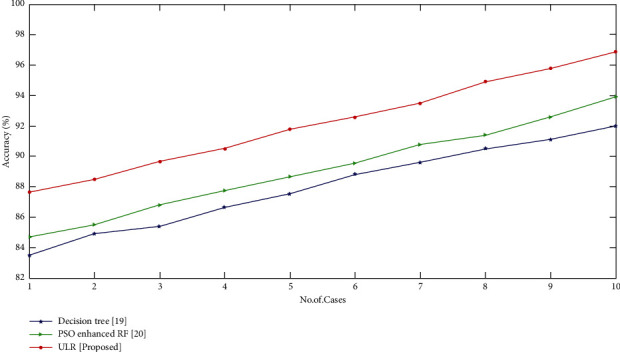
Comparison of accuracy (%) with proposed and existing techniques.

Figure 4 displays the comparison of accuracy (%) with existing and proposed approaches. Here, the quantity of dataset and accuracy are depicted. For each case, the accuracy of the proposed approach has nearly greater value than that of the existing approaches. Finally, we achieve our proposed approach with 96.7% accurateness of the ovarian tumor recognition.

## 5. Conclusion

This paper presents the ULR approach for clinical assessment of the ovarian tumors while pregnancy. The collection of 137 ovarian tumor cases was employed in the CEU test to divide these cases into benign and malignant tumors. Then, these images are preprocessed by WT technique for denoising procedure. Then, the features are removed by both LBP and LTE techniques to extract the characteristics of these images. In pregnant, the chances of being diagnosed with ovarian cancer are exceedingly slim, and the group of cases seems to be in first stage. For beginning phases of tumor, the general fetomaternal outcome was excellent. Patients experiencing ovarian malignancies discovered early in pregnancy decided to favor cancer therapy over their newborns, demonstrating that GA upon ovarian malignant detection is indeed a significant predictor in pregnancy results. The performance of the proposed technique was compared with conventional approaches to achieve our proposed approach with 96.7% accurateness. Future studies should guarantee appropriate randomization, concealment of the allocation sequence from participants and healthcare providers, blinding of outcome assessors to participants' treatment status, adequate follow-up, and histological identification of tumor recurrence to reduce the risk of bias. More study is needed to assess and validate the effectiveness of these and other future treatment options.

## Figures and Tables

**Table 1:** Clinical characteristics of 137 cases.

Clinical features	Quantity of cases
Age	
<36	97
≥36	40
Parity (n)	
0	78
≥1	59
Location	
UO	109
BO	28
Tumor diameter	
<7	26
≥7	111
GA of detection	
Prepregnancy	12
1st trimester	34
2nd trimester	39
3rd trimester	52
Symptoms	
Abdominal pain	23
Asymptomatic	114
GA of surgery	
1st trimester	25
2nd trimester	56
3rd trimester	51
Postpartum	5
Delivery mode	
Elective abortion	36
Transvaginal	12
CS	89
Pregnancy outcome	
Miscarriage	38
Preterm	27
Full-term	72

**Table 2:** ULR analysis of 137 ovarian cases.

ULR	Quantity of cases
BOT (*n* = 64)	
Serous	38
Mucinous	22
Endometrioid	2
Seromucinous	1
Other	1
EOC (*n* = 28)	
Serous	12
Mucinous	11
Primary peritoneal cancer	2
Clear cell	2
Brenner	1
MOGCT (*n* = 32)	
Immature teratoma	14
Dysgerminoma	16
Strumal carcinoid	2
MSCT (*n* = 4)	
Sertoli-Leydig tumor	2
Granulosa cell tumor	2
SCC (*n* = 2)	
Small cell carcinoma (hypercalcaemic type)	2
Metastatic carcinoma (*n* = 7)	
Krukenberg tumor	4
Cervix mixed adenocarcinoma	2
B-cell lymphoma	1

**Table 3:** Correlation of clinical characteristics in the abortion with live-birth groups.

	Abortion	Live birth	*P* value
Tumor diameter (cm), mean ± SD	13.89 ± 6.92	12.36 ± 7.68	>0.05
Reproductive history			> 0.05
Unipara	14	53	
Multipara	18	52	
FIGO stage			>0.05
1	22	78	
>1	10	27	
Surgical indication			>0.05
Emergency	6	12	
Select	26	93	
GA of detection			0.026
Prepregnancy	0	8	
1st trimester	22	42	
2nd trimester	10	55	
GA of surgery			0
1st trimester	21	2	
2nd trimester	11	19	
3rd trimester	0	82	
Postpartum	0	2	
Surgery			>0.05
Conservative surgery	8	48	
Fertility-sparing surgery	19	35	
Radical surgery	5	22	

## Data Availability

The datasets used and/or analyzed during the present study can be available from the corresponding author if needed.
